# NTyroSite: Computational Identification of Protein Nitrotyrosine Sites Using Sequence Evolutionary Features

**DOI:** 10.3390/molecules23071667

**Published:** 2018-07-09

**Authors:** Md. Mehedi Hasan, Mst. Shamima Khatun, Md. Nurul Haque Mollah, Cao Yong, Guo Dianjing

**Affiliations:** 1 School of Life Sciences and the State Key Lab of Agrobiotechnology, The Chinese University of Hong Kong, Shatin, Hong Kong; mehedicau@hotmail.com; 2 Laboratory of Bioinformatics, Department of Statistics, University of Rajshahi, Rajshahi 6205, Bangladesh; shammistat85@gmail.com (M.S.K.); mollah.stat.bio@ru.ac.bd (M.N.H.M.); 3 Department of Mechanical Engineering and Automation, Harbin Institute of Technology, Shenzhen Graduate School, Shenzhen 518000, China; yongc@hitsz.edu.cn

**Keywords:** post-translational modification, Wilcoxon-rank sum test, random forest, rule extraction

## Abstract

Nitrotyrosine is a product of tyrosine nitration mediated by reactive nitrogen species. As an indicator of cell damage and inflammation, protein nitrotyrosine serves to reveal biological change associated with various diseases or oxidative stress. Accurate identification of nitrotyrosine site provides the important foundation for further elucidating the mechanism of protein nitrotyrosination. However, experimental identification of nitrotyrosine sites through traditional methods are laborious and expensive. In silico prediction of nitrotyrosine sites based on protein sequence information are thus highly desired. Here, we report a novel predictor, NTyroSite, for accurate prediction of nitrotyrosine sites using sequence evolutionary information. The generated features were optimized using a Wilcoxon-rank sum test. A random forest classifier was then trained using these features to build the predictor. The final NTyroSite predictor achieved an area under a receiver operating characteristics curve (AUC) score of 0.904 in a 10-fold cross-validation test. It also significantly outperformed other existing implementations in an independent test. Meanwhile, for a better understanding of our prediction model, the predominant rules and informative features were extracted from the NTyroSite model to explain the prediction results. We expect that the NTyroSite predictor may serve as a useful computational resource for high-throughput nitrotyrosine site prediction. The online interface of the software is publicly available at https://biocomputer.bio.cuhk.edu.hk/NTyroSite/.

## 1. Introduction

Protein nitrotyrosination is a type of post-translational modification (PTM) that generally originates from an in vivo pathway involving highly reactive peroxynitrite (ONOO-) anion [1,2,3,4]. Excessive creation of nitric oxide (NO) and superoxide (O_2_^−^) by peroxynitrite anion has been indicated in aging and various diseases such as inflammation, hypoxia, and neurodegenerative disorders [2,5,6]. Occurred either within the protein catalytic sites or as part of the protein–protein interactive region, these modifications often decrease the electron intensity, negatively influence protein–protein interactions, and alter the diverse function of proteins [7,8,9]. Nitration can also arise within a tyrosine kinase phosphorylation motif to alter cellular signaling pathways [10]. Apart from these, how broadly the nitrotyrosination process is involved beyond modifications and what specific tyrosine site serve as nitroprotein in biological processes remain to be further investigated [2,11,12].

Accurate identification of nitrotyrosine sites is an essential foundation for revealing the mechanism and function of nitroproteins [13,14]. To identify nitrotyrosine sites, various large-scale proteomic studies are widely adopted in numerous organisms, including *Bacillus licheniformis*, *Bacillus subtilis*, *Bos taurus*, *Bungarus multicinctus*, *Capsella bursa-pastoris*, *Carica papaya*, Cricetulus *migratorius*, *Enterobacteria phage fd*, *Escherichia phage lambda*, *Enterobacteria phage T4*, *Enterococcus faecalis*, *Escherichia coli*, *Fusarium oxysporum*, *Halobacterium salinarum*, *Hirudo medicinalis*, *Homo sapiens*, *Mus musculus*, *Naja atra*, *Naja melanoleuca*, *Neurospora crassa*, *Ophiophagus hannah*, *Oryctolagus cuniculus*, *Ovis aries*, *Finegoldia magna*, *Phascolopsis gouldii*, *Physeter catodon*, *Pseudomonas putida*, *Rattus norvegicus*, *Rhodococcus rhodochrous*, *Saccharomyces cerevisiae*, *Schizophyllum commune*, *Staphylococcus aureus*, *Streptomyces albogriseolus*, *Struthio camelus*, *Sus scrofa*, *Thermus thermophilus*, *Tobacco mosaic virus*, and *Protobothrops flavoviridis* [1,7,8,10,14,15]. Despite the increasing number of experimentally identified nitrotyrosine sites, the mechanism of site-specific nitration modifications on tyrosine still remains largely unknown, possibly because of the constraints of measurement technologies [3,12,13]. On the other hand, experimental verification of nitrotyrosine sites is time-consuming, labor-intensive, and sometimes biased toward the abundant proteins. Therefore, an in silico approach can serve as an alternative strategy for proteome-wide identification of nitrotyrosine sites.

To date, several computational models have been proposed to predict nitrotyrosine sites [16,17]. The first predictor GPS-YNO2, by Liu et al. [17], was constructed from their original group-based prediction system (GPS) with four statistical measures (i.e., k-means clustering, weight training, matrix mutation, and motif length selection). Another predictor iNitro-Tyr, by Xu et al. [16], was based on a composition of pseudo amino acid encoding with a Jackknife test. The iNitro-Tyr and GPS-YNO2 predictors achieved a good performance on cross-validation (CV) tests using the training dataset (specificity at 85%, Matthews correlation coefficient at 49% and 21%, respectively). However, test on an independent dataset was not conducted.

In this work, nitrotyrosine and non-nitrotyrosine sites from nitrotyrosinated proteins were classified based on sequence evolutionary information features. To collect the protein evolutionary information, we first generated possible *k*-space amino acid pairs from the position-specific scoring matrix (PSSM), that is, the composition of profile-based of *k*-spaced amino acid pairs (pbCKSAAP), a method previously used for protein pupylation site prediction [18]. A non-parametric feature selection test, that is, a Wilcoxon rank-sum (WR) test, was then used to select informative and contributive features [19]. Meanwhile, a random forest (RF) classifier was assessed and a novel predictor NTyroSite was developed. The proposed method was rigorously benchmarked and the results demonstrated that it is highly competitive when compared with other existing tools using an independent dataset. Furthermore, the hidden and complex mechanism for nitrotyrosine site formation, comprehensible rules (i.e., feature combinations), and instructive features were extracted from the predictive RF model. NTyroSite is anticipated to serve as a useful tool with a confidence score for predicting nitrotyrosinated proteins and nitrotyrosine sites. In addition, we compared pbCKSAAP with three widely used sequence-based encoding methods, namely, composition of *k*-space amino acid pair (KSAAP), binary encoding (BE), and amino acid index properties (AAindex), through training datasets.

## 2. Materials and Methods

**Figure 1:**
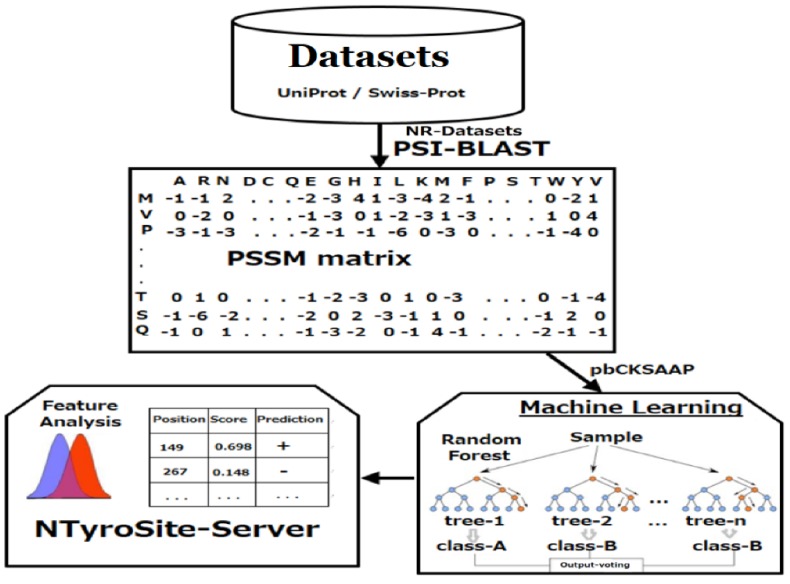
A computational framework of the proposed NTyroSite. NR—non-redundant; PSI-BLAST; PSSM—position-specific scoring matrix; pbCKSAAP—profile-based of *k*-spaced amino acid pairs.

In brief, after collecting non-redundant (NR) datasets, the PSSM was generated. Then, the sequence window was encoded by the pbCKSAAP scheme. Finally, the feature vectors were classified by RF to develop the NTyroSite predictor. A computational framework of the NTyroSite is shown in Figure 1.

### 2.1. Data Preparation

Selection of a suitable dataset for model development is the main challenge in predicting nitrotyrosine sites. The training data should ideally be derived from experiments. In this study, we collected 796 proteins with 1406 experimentally verified nitrotyrosine sites from the dbPTM, SysPTM2.0, and published articles in total [1,17,20,21]. After removing ≥40% sequence redundancy using CD-HIT [22], the same training dataset was retrieved from the GPS-YNO2 [17]. The training dataset includes 542 proteins with 1043 nitrotyrosine and 7612 non-nitrotyrosine samples. While the remaining 13 proteins with 19 nitrotyrosine and 201 putative non-nitrotyrosine sites were considered as an independent set.

Unbalanced datasets often make statistical learning procedures intractable and may hamper the accuracy [23,24]. To avoid possible biased prediction, non-nitrotyrosine sites were randomly selected from the whole negative sample pool to maintain a 1:1 positive versus negative sample ratio in the training model. The curated datasets are included in our online server.

Experimentally validated nitrotyrosine sites (tyrosine residues “Y”) were used as positive samples (i.e., nitrotyrosine sites), while all the remaining nitrotyrosine residues were considered as negative samples (i.e., non-nitrotyrosine) based on the intuitive assumption [25]. A sequence fragment of 2*w* + 1 length was used to represent each site with nitrotyrosine in the center. For the independent test, all the nitrotyrosine and non-nitrotyrosine sites were assessed to simulate the real situation.

### 2.2. Encoding Strategy of Sequence Evolutionary Information

To generate protein evolutionary information from the query sequences, a PSI-BLAST (version 2.2.26+) profile (NCBI NR90) database (December 2010) was directly used to generate the PSSM [26,27]. The e-value threshold cutoff and iteration times were set as 1.0 × 10^−4^ and 3, respectively. The *q_i_* (where *i* = 1, 2, …, 20) denotes 20 types of amino acid. Then, possible *k*-space amino acid pairs were calculated from the PSSM using *q_i_*{*k*}*q_j_*, that is, pbCKSAAP (where, *i*, *j* = 1, 2, …, 20). The collected peptide was reduced to dipeptide when *k* = 0. A dimension of (20 × 20) = 400 features (AA, AC, AD… YY)_400_ was composed for each value of *k*. In this study, the optimal *k_max_* was calculated as 4, indicating that 20 × (*k_max_* + 1) × 20 = 2000 differently spaced amino acid pairs were collected in each sequence fragments for computing the feature vector. For a sequence fragment with *L* residues, the corresponding PSSM has a dimensionality of *L* × 20. In the PSSM matrix, regarding an amino acid pair *q_i_*{*k*}*q_j_* that performs between positions *v* and *v + k +* 1, the composition score can be measured and normalized using the following equation:

(1) $$S_{ij}=\frac{\sum_{i,j=1}^{N}\max\left[\min\left\{\mathrm{PSSM}\left(v,q_{i}\right),\mathrm{PSSM}\left(v+k+1,q_{j}\right)\right\},0\right]}{L-k-1}$$

where *N* means *q_i_*{*k*}*q_j_* appears *N* times in the nitrotyrosine/non-nitrotyrosine site. PSSM (*v*, *q_i_*) denotes the score of residue *q_i_* at the *v*th row position of PSSM in *q_i_*{*k*}*q_j_*, and PSSM (*v* + *k* + 1, *q_j_*) positions for the residue *q_j_* at the (*v* + *k* + 1)th row position of PSSM in *q_i_*{*k*}*q_j_*. It is noteworthy that we also neutralized the gap (-) as a zero on the sequence fragments of nitrotyrosine/non-nitrotyrosine sites and did not count it in our final vector. More details of the pbCKSAAP scheme can be found in our previous article [18].

### 2.3. KSAAP Encoding

The KSAAP scheme is widely used for protein bioinformatics research [18,28,29]. The KSAAP scheme is described briefly as follows. If a fragment sequence is composed of 20 amino acids, it contains 400 (20 × 20) dimensional features (i.e., AA, AC, AD, …, YY)_400_ for every single *k* (*k* indicates the space between two amino acids). In this study, the optimal *k_max_* was set as 0–4 to generate a total of 2000 (20 × 20 × 5) dimensional feature vectors to each corresponding fragment sequence. The KSAAP encoding method was illustrated in our previous articles [18].

### 2.4. AAindex Encoding

In the AAindex database (version 9.1), the physicochemical properties of the amino acids were extracted [30]. After several trails, eight types of high-quality amino acid indices, including LIFS790101, TSAJ990101, NAKH920108, MAXF760101, BLAM930101, BIOV880101, CEDJ970104, and MIYS990104, were used. The indices were transformed into the nitrotyrosine and non-nitrotyrosine sequences to generate the numeric feature vectors. Values “NA” in the amino acid indices were replaced by (-) in this study. In sequence fragments through AAindex encoding, a 328-dimension (41 × 8 = 328) feature vector was generated.

### 2.5. Binary Encoding 

Each residue in the sliding window was generated by BE scheme and calculated 20-dimensional binary vectors [28]. An 820-dimension (41 × 20 = 820) feature vector was obtained via BE for a sequence. We measured 20 residues without counting any gap (-).

### 2.6. Random Forest

Random forest (RF) is an ensemble and supervised learning classifier [31,32,33] extensively used in bioinformatics research [28,34,35,36,37,38,39,40,41,42,43,44,45]. The RF works as a large collection of associated decision trees and the final classification is categorized by the votes from the entire trees. In this study, RF was used to classify the nitrotyrosine and non-nitrotyrosine samples. The following steps are involved in the construction of an RF. Firstly, to build a new random tree, a dataset with *m* variables at random from the *p* variables with replacement was prepared. Secondly, the best variable is selected among the *m* variables. Eventually, after collecting *n* decision variables, count the output votes by these variable and the classification is chosen based on the most votes. R package ‘RandomForest’ [46] was adopted to build our predictive model.

### 2.7. Rule Extraction

The predominant rules and informative features were extracted from the RF model to explain the prediction results. In a decision tree, from the root node to the leaf node, each path represents a rule. A straightforward method was adopted to extract the possible rules covering all the training data [28,34]. Firstly, consider the initial nitrotyrosine site rule set is empty and extract all the rules from the RF model that properly identify nitrotyrosine sites. Secondly, to assemble the rule that covers most nitrotyrosine sites, pick up the rule from the rule set of trained RF model and delete non-nitrotyrosine sites that adjust to this rule. Finally, repeat the second step until there are no non-nitrotyrosine sites in the training dataset. Similarly, the non-nitrotyrosine site rules can also be extracted.

### 2.8. Feature Selection

The rows of pbCKSAAP features represent the positive (+) and negative (−) windows generated from protein sequences. The columns of pbCKSAAP features represent the position specific residues. A new window of a protein sequence can then be classified into positive or negative windows based on the numerical score values of position specific residues, which are also known as features. Nonetheless, not all generated feature vectors can contribute equally to the classification model. Some redundant and uncorrelated information among the generated features can obstruct the prediction performance of a predictor. Feature selection methods are important in identifying crucial/relevant position-specific residue scores for classification. They can select useful features and advantage insights into inherent properties of protein sequences to avoid the overfitting problems and build a robust prediction model. Therefore, we consider a filtering approach to select the top differential features, which can make the significant contribution to the minimization of a misclassification error rate, for binary classification using RF classifier in this paper. The non-parametric WR test, which is equivalent to the Mann–Whitney *U*-test [19], is employed as an alternative to the two-sample *t*-test, in which observations are generated independently from two populations.

Assume *X*_1_, …, *X_N_* is a set of *N* sample observations. The method accessible here did not use the observations, but rather the number of ranks. The rank of *X_i_* among the *N* observations represented *R*(*X_i_*) = *X’_j_* ≤ *x_i_*. The testing procedures are the following:

(2) $$H_{0}\;:\;D_{1}\left(x\right)=D_{2}\left(x\right)$$

(3) $$H_{1}\;:\;D_{1}\left(x\right)\geq D_{2}\left(x\right)\;\mathrm{or}\;D_{1}\left(x\right)\leq D_{2}\left(x\right)$$

where *H*_0_ is the null hypothesis, which considers two samples are equal, while *H*_1_ is the alternative hypothesis. *D_j_*(*x*) is the distribution function for sample *j* = 1, 2, with 1 for nitrotyrosine and 2 non-nitrotyrosine sites. Moreover, we further investigated the minimum-redundancy–maximum-relevance (mRMR) [47] and information gain (IG) [48] feature selection for comparison with the WR scheme.

### 2.9. Performance Evaluation

To evaluate the performance of our prediction model NTyroSite, a 10-fold CV test was considered. Briefly, the training model was randomly and equally partitioned into 10 sub-folds. Among the 10 sub-folds, one fold was singled out as a test set, and the remaining nine folds were used as the training set. To benchmark the whole dataset, this process was repeated 10 times. Five necessary statistical matrices were measured, including accuracy (Ac), sensitivity (Sn), specificity (Sp), precision (Pr), and Matthews correlation coefficient (MCC). The following formulas were used to calculate these measures:

(4) $$Ac=\frac{n\left(TP\right)+n\left(TN\right)}{n\left(TP\right)+n\left(TN\right)+n\left(FP\right)+n\left(FN\right)}$$

(5) $$\mathrm{Sn}=\frac{n\left(TP\right)}{n\left(TP\right)+n\left(FN\right)}$$

(6) $$\mathrm{Sp}=\frac{n\left(TN\right)}{n\left(TN\right)+n\left(FP\right)}$$

(7) $$\mathrm{Pr}=\frac{n\left(TP\right)}{n\left(TP\right)+n\left(FP\right)}$$

(8) $$\mathrm{MCC}=\frac{n\left(TP\right)\times n\left(TN\right)-n\left(FP\right)\times n\left(FN\right)}{\sqrt{[\left(n\left(TN\right)+n\left(FN\right)]\times [n\left(TP\right)+n\left(FP\right)]\times [n\left(TP\right)+n\left(FN\right)]\times [n\left(TN\right)+n\left(FP\right)\right]}}$$

where *n*(*TP*), *n*(*TN*), *n*(*FP*), and *n*(*FN*) represent the numbers of true positives, true negatives, false positives, and false negatives, respectively. The scores of the statistical measurements (Ac, Sn, Sp, Pr) lie between 0 and 1 and MCC −1 to 1, and a higher score denotes a more accurate prediction. Furthermore, the receiver operating characteristics curve (ROC) (Sn vs. 1-Sp plot) was plotted and the area under an ROC (AUC) value was assessed using the pROC package in R [49,50].

## 3. Results and Discussion

### 3.1. The Difference of Amino Acid Propensity in Nitrotyrosine Protein

**Figure 2:**
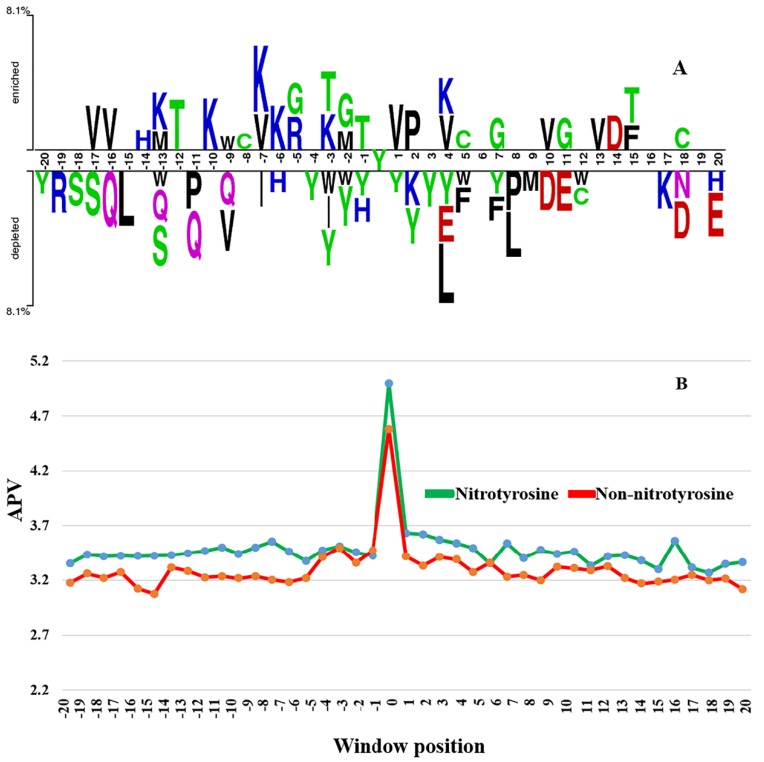
Comparison of sequence information between nitrotyrosine and non-nitrotyrosine sites. (**A**) A two-sample logo for training dataset of NTyroSite in compositional bias sequences of surrounding nitrotyrosined proteins; (**B**) we calculated the average PSSM value (APV) of each position (i.e., the average positions of each row of the PSSM matrix) in the flanking sequences of each nitrotyrosine (green color)/non-nitrotyrosine (red color) site, to investigate the protein evolutionary conservation of nitrotyrosine or non-nitrotyrosine sites. Because the optimal window size in this study was 41, the APVs of the positions [+1, +20] were averaged to obtain the APV of the downstream sites, while the APVs of the positions [−20, −1] were averaged to obtain the APV of the upstream sites. *p*-values were also calculated using the Kruskal–Wallis test and corrected using Bonferroni (See Table S1).

Firstly, to explore whether there is any significant difference in amino acid propensity between nitrotyrosine and non-nitrotyrosine sites, a sample logo was visualized (See Figure 2A) using Two Sample Logo software [51]. The amino acid residues that significantly enriched or depleted the surrounding nitrotyrosinated proteins were directly identified. A considerable difference in the surrounding fragment sequences was found between nitrotyrosine and non-nitrotyrosine sites. Specifically, in the enriched positions, residues ‘K, V, and G’ were more frequently observed; whereas in the depleted position residues, ‘Y and Q’ were more frequently observed. However, from the sequence logo, some sequence residues do not stack at the over- or under-represented positions of surrounding sequences (See Figure 2A). For instance, no stacked residues were found at the enriched positions of sequence logo “−20, −19, −18, −15, −11, −4, +3, +6, +8, +9, +12, +16, +17, +19, and +20” (See Figure 2A). Similarly, no stacked residues were detected in the depleted position of “−15, −14, −12, −10, −8, −7, −5, +6, +13, +14, +15, +16, and +19” (See Figure 2A). This result indicates that a frequency-based sequence encoding scheme is an efficient method to identify nitrotyrosine sites. Altogether, the above result suggests considerable sequence fragments difference between nitrotyrosine and non-nitrotyrosine sites, and pbCKSAAP is an effective method for nitroprotein prediction.

Secondly, we investigated the PSSM score difference between nitrotyrosine and non-nitrotyrosine sites’ sequence. The average PSSM value (APV) was used to describe the characteristic of the average conservation scores surrounding nitrotyrosine and non-nitrotyrosine sites. As shown in Figure 2B, the substantial difference in APV was found between nitrotyrosine (upstream) and non-nitrotyrosine (downstream) sites. Some of the neighboring residue positions neighboring nitrotyrosine sites were found considerably higher scores, particularly, for the upstream positions −16, −15, −12, −8, −1, +1, +6, +16, and +20 (See Figure 2B). The *p*-values were also calculated using the Kruskal–Wallis test and corrected by the Bonferroni scheme in surrounding nitrotyrosine and non-nitrotyrosine sites. As a result, we found that the *p*-value in some of the region was less than 0.05 (*p* < 0.05), as indicated with ‘*’ in Table S1, suggesting a significantly different distribution of amino acid residues between these two groups. The above results conclude that nitrotyrosine sites are more conserved (*p* < 0.05) in the upstream regions.

### 3.2. Prediction Performance of NTyroSite on the Cross-Validation Test

**Figure 3:**
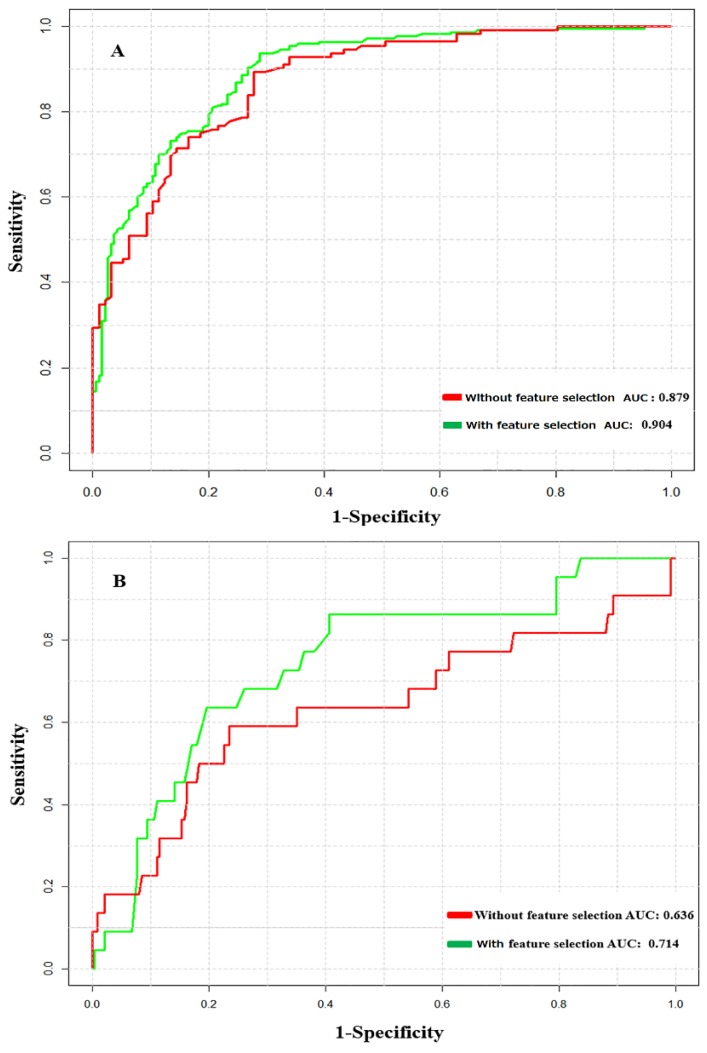
Performance comparison between without feature selection and with feature selection using receiver operating characteristics (ROC) curves: (**A**) performance based on 10-fold cross-validation (CV) test; (**B**) performance based on the independent dataset. AUC—area under an ROC curve.

A 10-fold CV test was performed based on the training dataset. Initially, the sequence fragments of nitrotyrosine and non-nitrotyrosine sites were encoded as numerical vectors using pbCKSAAP encoding (Materials and Methods). RF was grown as large as possible based on these features. The performance of models trained with different positive to negative samples ratios using 10-fold CV without feature selection approach is shown in Table 1. As seen, the number of negative samples showed more impact on prediction performance compared with that of positive samples. For instance, slightly decreased Sp, Sn, Pr, and MCC, and an increased Ac index were observed with the increase of negative sample numbers. Unbalanced datasets often pose a number of problems in classification accuracy of machine learning methods [28,29]. To avoid this problem, a positive to negative sample ratio of 1:1 was pooled to construct the final optimal predictive model of NTyroSite. The average performance of the NTyroSite scheme was summarized in Table 2. As seen, the performance index of Sp reached 90% (Sn = 0.616, Ac = 0.759, MCC = 0.537) without feature selection, and the performance was effective and eminently stable across all the cases. Meanwhile, in the ROC curve plot (See Figure 3A), the highest AUC value of the NTyroSite scheme was 0.879 in the training test. Moreover, the CV test for 4-, 6-, and 8-fold showed similar results, in which the model achieved AUC values of 0.859, 0.869, and 0.876, respectively. Furthermore, we calculated the performance across ten sets using a 10-fold CV test, from the random sampling of positive versus negative ratio 1:1 dataset. Table S2 showed that the prediction performance on different random sampling on the training test did not have much difference and the NTyroSite predictor provides a stable performance.

To further validate the robustness and performances of NTyroSite, we combined the training and independent sets and analyzed again via 3-fold CV tests using the randomly sampled datasets with a different positive versus negative ratio (See Table S3). Although the performances yielded are slightly lower than the original training model of NTyroSite, the consistent performance still proved the robustness of NTyroSite in predicting nitrotyrosination proteins.

The local window size is an important factor for PTMs’ site prediction [18]. In this study, the window sizes were optimized based on AUC values using a 10-fold CV test. To assess the sequence similarity of the long-range region around the nitrotyrosine sites, the windows size was increased from 13 to 45 for without and with feature selection approach (See Figure S1). Furthermore, *p*-values were also calculated using the Kruskal–Wallis test and corrected using Bonferroni. We found that the *p*-value in some of the window positions was less than 0.05 (*p* < 0.05), suggesting that the performance was substantially improved with feature selection approach (See Figure S1). An optimal window size of 41 (−/+20) was finalized after several trials. It is noteworthy that fewer numbers of residues included in a smaller window size might result in loss of potentially useful information [52], which is the main reason why we chose to use a larger window size.

### 3.3. Analysis of Feature Optimization Results Using Wilcoxon Rank-Sum Test

The contribution of each feature was analyzed using the WR test, a novel feature selection algorithm for protein bioinformatics research. The WR scheme significantly reduces the dimensionality of the high dimensional pbCKSAAP features, and the top 200 features were collected as optimal feature vectors. Meanwhile, these feature vectors were reconstituted into a new orderly feature, which was then applied to build the final prediction model of NTyroSite. As shown in Table 2, NTyroSite achieved a much better performance with feature selection. For training set performance, the MCC scores increased to approximately 7% when Sp was controlled at 90% (See Table 2). The depicted ROC curves show that the AUC value increased to approximately 3% with feature selection on the training set (See Figure 3A). For an independent dataset, the AUC value increased approximately 8% with feature selection (See Figure 3B). With feature selection approach, the performances of models using different training and independent test datasets are shown in Figure S2. The above result shows that the WR method could effectively extract those valuable dimension vectors from the pbCKSAAP features.

**Figure 4:**
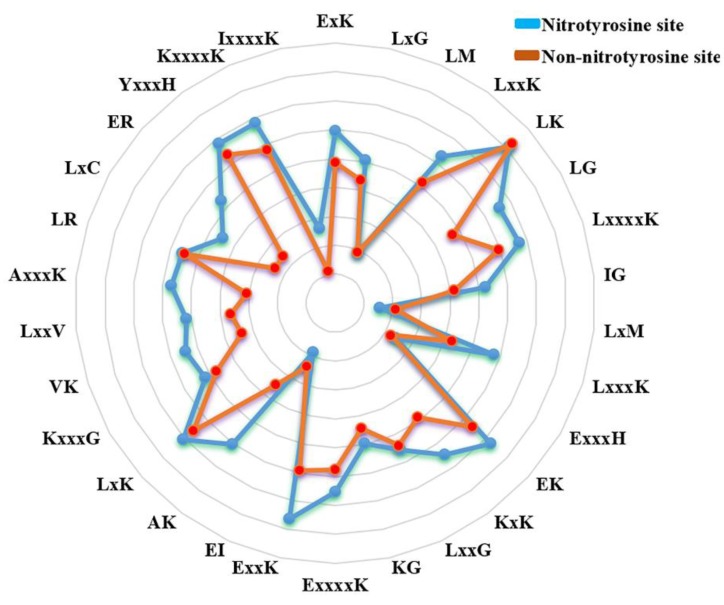
Top 30 amino acid residue pairs selected by the Wilcoxon rank-sum (WR)-based feature selection from the pbCKSAAP scheme. Blue color denotes nitrotyrosine sites and dark red color denotes non-nitrotyrosine sites. The radar diagram is represented by the composition of each residue pair whose length is proportional to the composition of pbCKSAAP features.

In addition, to examine the most significant residues and positions surrounding nitrotyrosine and non-nitrotyrosine sites, 30 top-ranked amino acid pairs were collected using a WR feature selection approach. These important features were visualized in a radar diagram (See Figure 4). The feature ‘E×K’ was characterized by 1-spaced residue pair of ‘EK’, where ‘×’ standpoints for any amino acid. The feature ‘E×K’ was the most important residue pair, demonstrating the most enhanced motif nearby nitrotyrosine sites. Likewise, the feature ‘LM’, representing a 0-spaced residue pair of ‘LM’, is also enriched in the nitrotyrosine sites. The same illustration applies to the other *k*-space residue pairs. It is intriguing that most of the top 30 features contain aliphatic and basic residues, such as “G, A, V, I, L” and “H, K, R”, respectively (See Figure 4), which may perform in the recognition of nitrotyrosine sites. We also noticed that the possible all *k*-space residue pairs of the pbCKSAAP scheme, such as (“ ”, “×”, “××”, “×××”, “××××”), were involved in the top 30 significant features (See Figure 4), indicates that all space residue pairs are important, and together they make a mutual contribution to the prediction of nitrotyrosine sites.

Previous studies indicate that protein tyrosine nitration is a discriminating process [53]. Peptide mapping studies specified that three main factors may determine which tyrosine residues in a protein is specially nitrated, including nitration mechanism, protein structure, and environment where the protein is located [54,55]. Particularly, some general tendencies include preferred nitration in tyrosine’s situated near charged residues or on a loop structure, and nearby cysteine residues may constrain tyrosine nitration [39]. However, none of them are conclusive.

Our studies have provided the evidence that the pbCKSAAP predictor is able to characterize the hidden and complex conserved motifs in the nearby nitrotyrosine sites. Indeed, as shown in Figure 4, in the list of 30 most-significance features, the possible combination of all *k*-spaced amino acid pairs, aliphatic, and basic residues could be found. Here, three of the most significant aspects of this scheme could be highlighted. The first one is the usage of composition *k*-spaced amino acid pair to reflect the coordinated pairs of residues near the nitrotyrosine sequence motif. Secondly, the analysis result showed that several flanking positions of the nitrotyrosine sites were more conserved than the corresponding positions of non-nitrotyrosine sites (See Figure 2B). As importantly, pbCKSAAP surrounds the evolutionary features information into its encoding from the PSSM profile. Altogether, these results demonstrate that the composition of all possible *k*-space residue pairs could serve as an important sequence feature to represent protein nitrotyrosine patterns.

### 3.4. Performance Comparison with Existing Tools on the Test Dataset

Currently, only two computational predictors, namely, GPS-YNO2 and iNitro-Tyr, employ different training datasets to predict nitrotyrosine sites [16,17]. The proposed NTyroSite predictor was therefore compared with these two predictors using an independent dataset (Materials and Methods). We submitted the independent dataset to GPS-YNO2 and iNitro-Tyr online servers and evaluated the performance. The NTyroSite predictor achieved a much better performance than GPS-YNO2 and iNitro-Tyr in terms of Ac, Sn, Sp, Pr, and MCC (See Table 3). The proposed scheme MCC score was significantly higher than that of the GPS-YNO2 and iNitro-Tyr predictors (See Table 3). Interestingly, the NTyroSite and other two existing predictors showed a significantly lower Pr value, possibly due to the highly imbalanced independent dataset used. In Table 1, the Pr value was also significantly decreased with the increase of negative sample in CV test.

The proposed NTyroSite outperformed the GPS-YNO2 [17] and iNitro-Tyr [16] predictors. The GPS-YNO2 mainly measured the positional sequence encoding (i.e., this schemes depicted the positional amino acid properties using the position along the sequence fragments), including k-means clustering, matrix mutation, weight training, and motif length selection to predict the nitrotyrosine sites. Similarly, Nitro-Tyr was a positional amino acid encoding based predictor (i.e., pseudo amino acid composition). The proposed NTyroSite has encoded amino acid frequency based method with protein evolutionary information (i.e., initially proposed model encoded positional PSSM from the sequence, and then amino acid possible pairs were calculated from the PSSM). Moreover, some amino acid residues are not over-/under-represented in the specific sequence positions (See Figure 2A), which suggests that the amino acid frequency encoding was an effective scheme to classify the nitrotyrosine sites (see details in the previous section). In addition, the authors of GPS-YNO2 and iNitro-Tyr predictors did not select any independent test dataset to check their training models. In doing so, these predictors might somehow be biased and the resulting performance is not satisfying in our independent testing set.

Furthermore, 375 nitrated proteins were collected from PubMed (See Table S4) as a showcase application. Although these *bona fide* nitrated tyrosines are yet to be verified experimentally, our model successfully annotated 322 (86%) potential proteins with at least one nitrotyrosine site. These analyses and prediction results can be conveniently used for future experimental investigation.

### 3.5. The Influence of Peptide Level Sequence Redundancy on the Predictive Model

**Figure 5:**
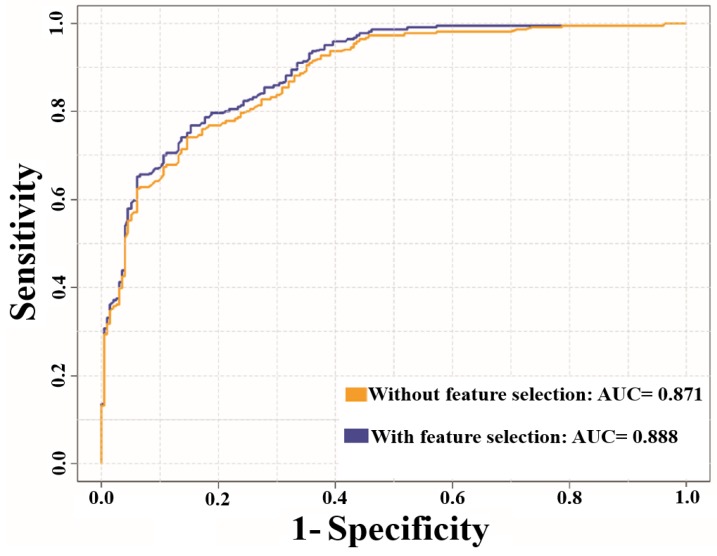
AUC values after sequence redundancy removal. Blue and orange color represents without and with feature selection.

We used an in-house PERL script to eliminate the redundant nitrotyrosine/non-nitrotyrosine sites (with 40% amino acid identity cutoff). After removal of the peptide level redundancy, a training dataset (951 nitrotyrosine and 6718 non-nitrotyrosine sites) and a testing dataset (17 nitrotyrosine and 183 non-nitrotyrosine sites) were re-assembled. From the training dataset, positives to negatives ratio of 1:1 were pooled out. The overall prediction of NTyroSite in the 10-fold CV decreased slightly (AUC = 0.871) after removal of the redundant sequences (See Figure 5). Similarly, the performance was decreased slightly (AUC = 0.888) with feature selection. For the independent test, NTyroSite predictor still achieved the best performance when compared with GPS-YNO2 and iNitro-Tyr predictors after redundancy removal (See Table S5).

### 3.6. Rules Extracted from NTyroSite Model

To dissect the NTyroSite prediction model, a rule extraction scheme was adopted (Material and Method section). The top 10 important rules were extracted from the trained NTyroSite model to examine the effects of different features on the pbCKSAAP encoding scheme. Table 4 gives a full description of each specific rule and the number of nitrotyrosine sites covered by the corresponding rule. For the individual rule, *I*(*·*) indicates the frequency of the pattern and “&” represents the logical conjunction. As shown, some amino acid pairs, for example, ‘*A××××K, R××K, K×××A, S××××G, LG*, and *G××H*’, frequently co-occurred in the top 10 rules. This specifies that the amino acid propensities in the corresponding positions are associated with the nitrotyrosine sites. The aliphatic and basic residues, such as, “G, A, V, I, L” and “H, K, R”, were also found in the top 10 rules extracted from the NTyroSite model (See Table 4). In Figure 4, aliphatic and basic residues were also observed in the top 30 features. Therefore, we deduce that aliphatic and basic residues may influence the recognition of nitrotyrosine sites. Taken together, the efficient classification tool RF is particularly powerful in explaining the prediction results for a trained model.

### 3.7. Case Studies

An RF model was built to evaluate the performance of the proposed model, using the selected 200 features on the training dataset. Two experimentally identified independent proteins, that is, *Homo sapiens* (PDB ID: 2ZGV, Chain A) [56] and the metal binding protein in human (PDB ID: 4UPG, chain: A) [57], were randomly examined under a realistic condition. These two proteins are involved in a variety of biological processes such as treatment of cancer, viral infections, endoplasmic reticulum stress, and cell death. The probability of nitrotyrosine sites (Y76, Y161, Y196, Y383, and Y116) were 0.827, 0.881, 0.888, 0.473, and 0.654, respectively, as given by the proposed NTyrosite server. The NTyroSite predictor was identified as four TP among the five nitrotyrosine sites. To demonstrate these prediction results, the best rules can also be extracted from the NTyroSite model to detect nitrotyrosine sites. As listed in Table 4, the best rules covered by NTyroSite are as follows: *I(A××××K) > 0.118* and *I(R××K)* ≤ *0.089* and *I(L×××M)* ≤ *0.069* and *I(A××R)* ≤ *0.068.* In this rule, *I(np)* indicates the frequency of profile-based amino acid pair *np* for pbCKSAAP.

### 3.8. Comparison of WR with IG and mRMR Feature Selection Scheme

Moreover, we compared the WR scheme with IG and mRMR using the top 200 training features via a 10-fold CV test. The models’ performances with feature selection approach were shown in Table 5. The results showed that the performance of IG and mRMR were slightly decreased with feature selection implemented (See Table 5). The WR scheme could effectively extract those valuable dimension vectors from the pbCKSAAP features in nitrotyrosine prediction compared with IG and mRMR schemes.

### 3.9. Comparison with Different Sequence-Based Features

To further evaluate the performance of pbCKSAAP, three well-encoded sequence-based features of KSAAP, AAindex, and BE encodings were examined by the training dataset. The final model of NTyroSite used a 1:1 positive versus negative ratio for training datasets. Therefore, we used the same training dataset via a 10-fold CV test. The performance of the three encoding methods is shown in Table 6. As seen, the proposed pbCKSAAP yielded a more competitive performance compared with AAindex, BE, and KSAAP methods. The performance comparison indicated that the encoding scheme of pbCKSAAP is useful and powerful for nitrotyrosine sites prediction.

### 3.10. Web Server

To serve the potential user, an accessible online server of NTyroSite (nitrotyrosine site predictor) was developed and is available at https://biocomputer.bio.cuhk.edu.hk/NTyroSite/. The web server was made using some language programming such as Perl, CGI scripts, PHP, HTML, and CSS. In the index page, users can submit their query sequence by browsing their own file using browse button or pasting it into the text box. The server will initially generate tyrosine fragments of all the putative nitrotyrosine sites for a query protein. Meanwhile, by performing the PSI-BLAST search, the server will generate the PSSM for the query protein and calculate the possible *k*-space amino acid pair. After collecting the contributive features using the WR method, the server will classify the sequence similarity with the assistance of an RF classifier. The server will return the result consisting of the job ID, query protein name, residue positions, and RF scores of the predicted nitrotyrosine sites on the output webpage. The server will create a job ID such as “20160102900067” and user can reserve this ID for future query. Note that the NTyroSite server only takes in FASTA format sequence.

## 4. Conclusions

In this paper, we developed an in silico tool NTyroSite for protein nitrotyrosine sites prediction. Benchmarking experiments based on CV and independent tests demonstrated that NTyroSite achieved a promising and competitive performance compared with several existing predictors. Moreover, we carried out a feature selection and rule extraction methods to facilitate the better understanding and interpretation of our prediction model. Finally, a user-friendly web server was implemented to the research community, which is freely accessible at https://biocomputer.bio.cuhk.edu.hk/NTyroSite/.

## Figures and Tables

**Table 1:** The performance of the proposed models trained with different positive versus negative samples ratios based on 10-fold cross-validation (CV) test. MCC—Matthews correlation coefficient.

The Ratio (P/N)	Sp	Sn	Pr	Ac	MCC
1:1	0.901	0.616	0.865	0.759	0.537
1:2	0.900	0.567	0.757	0.789	0.511
1:3	0.902	0.548	0.612	0.813	0.474
1:total	0.904	0.523	0.438	0.856	0.416

**Table 2:** NTyroSite prediction performances for without and with features selection.

Measurement	Training Test		Independent Test	
	without Feature Selection	with Feature Selection	without Feature Selection	with Feature Selection
Sp	0.901	0.900	0.806	0.801
Sn	0.616	0.675	0.479	0.609
Pr	0.865	0.884	0.197	0.231
Ac	0.759	0.787	0.778	0.782
MCC	0.537	0.601	0.196	0.272

**Table 3:** Comparison of NTyroSite with existing predictors using an independent test set.

Measurement	GPS-YNO2	iNitro-Tyr	NTyroSite
Sp	0.791	0.796	0.801
Sn	0.211	0.211	0.609
Pr	0.087	0.089	0.231
Ac	0.741	0.745	0.782
MCC	0.002	0.004	0.274

The threshold values of GPS-YNO2 is considered as medium. However, the threshold value iNitro-Tyr is consistent with value defined in the server. And the proposed NTyroSite predictor threshold is controlled, the same as at Sp 90% of training model performances.

**Table 4:** The extracted rules collected from NTyroSite model.

No.	Individual Reports of Rule Extraction	No. of Samples Covered by Rule
1	*I(K* × *L) > 0.197*	172
2	*I(GK) > 0.113 & I(K××××L)* ≤ *0.097 & I(ST)* ≤ *0.038*	112
3	*I(A××××K) > 0.118 & I(H××××A)* ≤ *0.013*	90
4	*I(YG)* ≤ *0.068 & I(K×××A)* ≤ *0.014 & I(S××××G)* ≤ *0.039 & I(R××K)* ≤ *0.089 & I(V×××M)* ≤ *0.014 & I(E××C)* ≤ *0.038 & I(EV) > 0.163*	78
5	*I(R××K)* ≤ *0.089 & I(L××××H)* ≤ *0.118 & I(S*×*K)* ≤ *0.092 & I(SR) > 0.229*	67
6	*I(A××R)* ≤ *0.068 & I(K×××A)* ≤ *0.014 & I(S××××G)* ≤ *0.039 & I(R××K) > 0.089 & I(V*×*R)* ≤ *0.026*	49
7	*I(A××××K)* ≤ *0.118 & I(R××K)* ≤ *0.089 & I(LG)* ≤ *0.113 & I(G××H)* ≤ *0.095 & I(EM)* ≤ *0.013 & I(K××××G)* ≤ *0.042 & I(G*×*A) > 0.141*	47
8	*I(G××H) > 0.149 & I(L×××M)* ≤ *0.069 & I(DG)* ≤ *0.038 & I(Y×××K)* ≤ *0.0139 & I(A×K)* ≤ *0.034*	44
9	*I(Y*×*H) > 0.163 & I(V×R)* ≤ *0.013 & I(LG)* ≤ *0.113 & I(K×××I)* ≤ *0.128*	39
10	*I(DK)* ≤ *0.092 & I(G×××G) > 0.122 & I(S×K)* ≤ *0.013*	35

For each rule, *I*(*n*, *w*) indicates the amino acid *n* at position *w* and ‘‘&’’ denotes the logical conjunction and.

**Table 5:** Comparison with different feature selection schemes. IG—information gain; mRMR—minimum-redundancy–maximum-relevance; WR—Wilcoxon rank-sum.

Methods	IG	mRMR	WR
Sp	0.897	0.899	0.900
Sn	0.601	0.596	0.675
Pr	0.861	0.855	0.884
Ac	0.749	0.748	0.787
MCC	0.511	0.507	0.601

**Table 6:** Performance comparison with different sequence-based features. AAindex—amino acid index; BE—binary encoding; pbCKSAAP—profile-based of *k*-spaced amino acid pairs.

Methods	Sp	Sn	Pr	Ac	MCC
AAindex	0.896	0.442	0.802	0.669	0.424
BE	0.899	0.435	0.799	0.667	0.402
KSAAP	0.900	0.587	0.857	0.744	0.501
pbCKSAAP	0.901	0.617	0.865	0.759	0.538

## Supplementary Materials

The following are available online: Figure S1–S2; Table S1–S5.
