# Supplementary material for: NTyroSite: Computational Identification of Protein Nitrotyrosine Sites Using Sequence Evolutionary Features
Source: Molecules. 2018 Jul 9;23(7):1667. doi: 10.3390/molecules23071667 (PMC6099560; doi:10.3390/molecules23071667)
Supplement: Supplementary file 1 [file molecules-23-01667-s001.pdf]

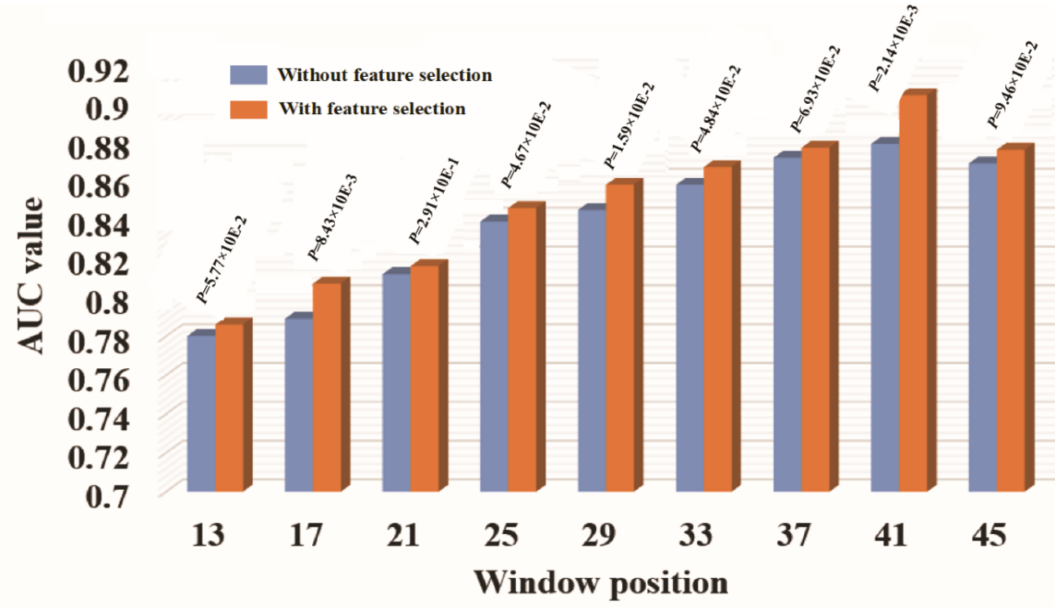

Figure S1 Area under a receiver operating characteristics curve (AUC) values based on different window sizes. Blue and orange color represents without and with feature of selection. *p*-values were also calculated using the Kruskal–Wallis test and corrected by Bonferroni scheme.

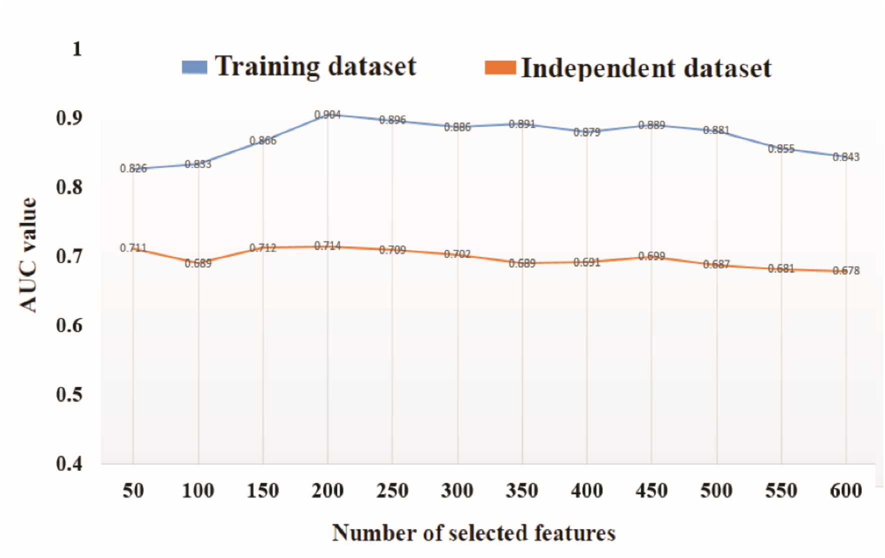

Figure S2 The model performances using different training and independent datasets with Wilcoxon-based feature selection approach.

Table S1 *p*-values were calculated using the Kruskal–Wallis test and corrected using Bonferroni of surrounding nitrotyrosine sites and non-nitrotyrosine sites. '*' represents p-value < 0.05.

Positions	*p*-value	Positions	*p*-value
-20	1.00	0	4.49E-02*
-19	9.01E-01	1	5.88E-02
-18	1.00	2	1.73E-02*
-17	8.18E-01	3	1.00
-16	4.74E-01	4	1.00
-15	2.15E-02*	5	4.43E-02*
-14	1.46E-02*	6	1.00
-13	1.00	7	3.27E-02*
-12	1.00	8	1.00
-11	4.76E-02*	9	3.22E-02*
-10	4.14E-02*	10	1.00
-9	3.01E-02*	11	1.00
-8	1.34E-02*	12	1.00
-7	7.23E-02	13	3.29E-02*
-6	1.82E-02*	14	1.19E-02*
-5	1.00	15	6.29E-02
-4	1.00	16	1.00
-3	1.00	17	2.67E-03*
-2	1.00	18	1.00
-1	1.00	19	1.00
		20	1.00

Table S2 The performance across ten sets using 10-fold cross-validation test, from the random sampling of positive versus negative ratio 1:1 dataset. MCC—Matthews correlation coefficient; AUC—area under a receiver operating characteristics curve.

Samples	Sp	Sn	Pr	Ac	MCC	AUC
1	0.901	0.617	0.865	0.756	0.538	0.879
2	0.902	0.664	0.886	0.783	0.578	0.887
3	0.899	0.650	0.871	0.774	0.565	0.884
4	0.893	0.630	0.860	0.761	0.542	0.880
5	0.894	0.626	0.862	0.760	0.533	0.875
6	0.897	0.611	0.861	0.754	0.527	0.871
7	0.888	0.593	0.843	0.740	0.496	0.863
8	0.900	0.674	0.880	0.787	0.587	0.891

9	0.895	0.645	0.867	0.770	0.557	0.881
10	0.892	0.612	0.851	0.752	0.522	0.869
Average	0.896 ±0.005	0.632±0.026	0.865±0.013	0.764 ±0.015	0.545±0.0276	0.878±0.009

* ±, represents the mean standard variance.

Table S3. The performance of the combined of training and independent sets with different positive versus negative samples based on a three-fold cross-validation (CV) test.

The Ratio (P/N)	Sp	Sn	Pr	Ac	MCC
1:1	0.893	0.596	0.859	0.745	0.518
1:2	0.897	0.563	0.753	0.786	0.489
1:3	0.900	0.530	0.558	0.801	0.468
1:total	0.901	0.509	0.432	0.851	0.403

Table S4 From large-scale and small-scale experimental studies, 375 potentially nitrated proteins were collected, although they are still not experimentally determined. The default threshold of NTyroSite was adopted.

UniProt ID	NTyroSite predicted sites	Organisms	PMID
O15438	962,978,1490	H. sapiens	15695394;18922976
O60674	42,44,201,206,221,449,570,868,966,972	H. sapiens	19635391
O95169	150,161,163,167	H. sapiens	19897030
P00533	270,275,285,299471,626,727,978,1092	H. sapiens	9822654
P00738	346,352,386,389	H. sapiens	1154943
P01019		H. sapiens	9578472;10518791
P01574	176	H. sapiens	678325
P02671	737,740,851,	H. sapiens	12189015
P02679	27,44,389,403	H. sapiens	12189015
P02730	299,413	H. sapiens	9409547
P04075	3,5,174,204,214,223,302,328,343,364	H. sapiens	14684358;15345482;20511553
P04150	660,663	H. sapiens	2960354
P04406	42,45,49,94,255,276,314,320	H. sapiens	12675516;11593016;16378731
P05091	131,473,485,497,502	H. sapiens	14684358
P07437	36,50,51,59,106,222	H. sapiens	14684358
P08107	371,545	H. sapiens	14684358
P08185		H. sapiens	7066412
P08574	174,179	H. sapiens	12516089
P09104	44,57,131,189,200,236,252,257,270	H. sapiens	12787059

P12814	161,241	H. sapiens	16492779;19895807
P13500	512,515,569	H. sapiens	10484461
P13569	1307	H. sapiens	12194970
P16112	192,211,287,309,517,521,536,611,622,745	H. sapiens	14715284
P17661	54,388,405	H. sapiens	17882015
P18031		H. sapiens	10486138
P21399	184,185,197,466,666,695,759,774,800,835	H. sapiens	12820873
P21796	7,22,118,146,153,195,247	H. sapiens	14684358;16378731
P22301		H. sapiens	12370394
P22626	276,283	H. sapiens	14684358
P23141	386,403,445,447,451	H. sapiens	12516089
P25445	321,337,352	H. sapiens	14679192
P27169		H. sapiens	18682903
P35222		H. sapiens	17030184
P35354	134	H. sapiens	9202025
P41240	18,48,64,214,380	H. sapiens	10845713
P42224		H. sapiens	16250053
P42330	24,55,81,114,196,259,272	H. sapiens	14684358
P42574	274,276	H. sapiens	12516089
P49908	205	H. sapiens	9792455
P56945	12,192,327	H. sapiens	10401986
P60174	16,85,202	H. sapiens	12787059;19251756
P68366	103,108,161,262,272,282,357	H. sapiens	14684358
Q05940		H. sapiens	18599602
Q06323		H. sapiens	14684358
Q09472	468,475,611,1503,1689,1886	H. sapiens	1053046
Q13011	149	H. sapiens	14684358
Q8MGQ7	31,51,221	H. sapiens	18764882;19651177
Q92769	3,88,253	H. sapiens	15013452
Q99426	28,57,191,197,213,220	H. sapiens	18048340
Q99571	54,219,292,299,300,315	H. sapiens	16793133
Q99798	151,390,472	H. sapiens	14684358
A2AQ25	289,435,837,987,1061,1260	M. musculus	19150419
O08677	54,72,76	M. musculus	17615369
O08709	89,217,220	M. musculus	16622028;19150419
O08749	54,351,386,473	M. musculus	19150419
O22769	114,125,186	M. musculus	12821649
O35074		M. musculus	17065353

O35639	15,136,183	M. musculus	16622028
O35744	336	M. musculus	16622028
O35945	119,457	M. musculus	16622028
P01027	244,354,634,1027,1261,1262,1266,1348,1392	M. musculus	17615369
P01731	82,196,219	M. musculus	17603493
P01867	8,31,292,295,369,396	M. musculus	17615369
P01873	65,440,454	M. musculus	17615369
P06151	83,145,239,247	M. musculus	16622028
P06909	1146,1147	M. musculus	17615369
P07309	30,188,189	M. musculus	17615369
P07356	269,275	M. musculus	16622028
P08074	149	M. musculus	16622028
P08226		M. musculus	17615369
P09103	45,51,65,96,101,118,395,459	M. musculus	16622028
P09542	82	M. musculus	19150419
P09671	58,69,189,200,217	M. musculus	12821649;16622028;16651627;19129394
P10107	39,207,283	M. musculus	16622028
P10300	115,195,197	M. musculus	17603493
P10639	80	M. musculus	17561092
P11103	52,309,310,638,644,991,1000	M. musculus	18829681
P15208	171,259,272,273,534,539,657,660	M. musculus	19682478;20103705
P15626	7,23,28,33,62,79,138,161	M. musculus	16622028
P17182	131,189,200,236,252,257,270,	M. musculus	15378736
P17563	12,28	M. musculus	16622028
P20152	53,61,276,383	M. musculus	10606512;19956584
P22599		M. musculus	17615369
P24529	265,314,371,389,423,448	M. musculus	9636206
P26443	53,170,193,319,439,451,458,464,528,539,550	M. musculus	19150419
P27773	67,95,100,115,278,445,454,467	M. musculus	16622028
P28271	197,695	M. musculus	15258160
P28867	12,64,155,187,372	M. musculus	16314418
P31750	38,175,176	M. musculus	19682478;20103705
P32233	94,108,288,295,305,313	M. musculus	16622028
P35569	489,628,658,935,983,1006	M. musculus	19682478;20103705
P37238	173	M. musculus	12163159
P40936	177,243,247	M. musculus	16622028
P42225		M. musculus	11159690
P46638	8,73,81,112,132	M. musculus	19150419

P51881	187,191,195,251,291,297	M. musculus	19272377
P62334	386	M. musculus	19597039
P63085	62,126,129,137	M. musculus	18523870
P70288	3,88,304	M. musculus	19164702
P70313	162,209,216,250,253	M. musculus	11316578
P81122	111,116,621,624	M. musculus	20103705
Q05769	134	M. musculus	16375865
Q07100	311	M. musculus	18791203
Q60932	20,35,208,238,260	M. musculus	12821649;19150419
Q61292	397,900,921	M. musculus	19956584
Q61646	287,293	M. musculus	17615369
Q61838	98,136,829	M. musculus	17615369
Q62120	42,44,206,221	M. musculus	17510231;17510232
Q63844	82,146,149,157	M. musculus	18523870
Q6PF93	9,11,35	M. musculus	9826526
Q6YK32	68	M. musculus	17615369
Q8CGI9	96	M. musculus	19150419
Q8K0E8	312,346,398,424,442	M. musculus	17615369
Q8K4Z3	70,93,251	M. musculus	17615369
Q8R4K2	201	M. musculus	20304961
Q8VCM7	26,269,303,318,402	M. musculus	17615369
Q91X72	197,198,219	M. musculus	17615369
Q91XX4	424	M. musculus	19150419
Q91Y86	26,44,71,185,190	M. musculus	18523870
Q91ZJ5	298,442	M. musculus	19150419
Q921I1	104,113,114,115,338,429,430	M. musculus	17615369
Q99JY0	301,332,337,343	M. musculus	12821649
Q99K47	18,23,26,34,36,142	M. musculus	17615369
Q99KQ4	230,231,341,403	M. musculus	19150419
Q99MN9	338,407	M. musculus	19150419
Q9CR16	115	M. musculus	19272377
Q9CR68		M. musculus	19150419
Q9DCT2	165,244	M. musculus	19150419
Q9EQ20	443	M. musculus	19150419
Q9ER56	60,185,192	M. musculus	15378736
Q9JJV2	30,99,141	M. musculus	16622028
Q9JK42	359,374,382	M. musculus	16622028
Q9Z1E3	181,195,302	M. musculus	15464059

P00637	165,168	O. cuniculus	2553704
P00949	322,353	O. cuniculus	41237
P46409	62,79,197	O. cuniculus	12620373
Q08862	9,74,82	O. cuniculus	12620373
Q08863	132,165	O. cuniculus	12620373
Q2QN81	230,356	O. sativa	19717529
Q67TZ4	43,99	O. sativa	19717529
Q75L89	361,371	O. sativa	19717529
Q8W314	151,387	O. sativa	19717529
P42272	329,331	P. mirabilis	241412
P42273	116,141	P. mirabilis	241412
P10933	104	P. sativum	19717529
Q9SC31	268,277	Populus	19717529
P02879	150,158,229,462	R. communis	7470048
P00799	267	R. miehei	4433573
A7VJC2	276,283,306	R. norvegicus	11593016;19402213
B2RZD6		R. norvegicus	15345482
O08557		R. norvegicus	18072209
O09171	77,128	R. norvegicus	11593016
O74200		R. norvegicus	17254026;18072209;19402213
P00507	67,75,96,316	R. norvegicus	11593016;19402213
P02600	124,179	R. norvegicus	15345482
P02770	108,162	R. norvegicus	18072209
P04182	55,69,78	R. norvegicus	18072209
P04636	161,253	R. norvegicus	15345482;17254026;19402213
P04762	280,308	R. norvegicus	11593016;18072209
P04905	23,28,33,4116	R. norvegicus	11593016
P05504		R. norvegicus	11593016
P05982	105,127,129	R. norvegicus	11593016;15345482
P06686	9,146,147	R. norvegicus	17254026
P07323	44,57,131,189,200,236,252,257	R. norvegicus	18072209
P07335	39,68,82,100,269,279	R. norvegicus	17254026
P07340	68,167,169,205,280,286	R. norvegicus	17254026
P07379	140,235,488,493	R. norvegicus	18072209
P07824	45,176,188	R. norvegicus	506610
P07895	58,59,189,200,217	R. norvegicus	15327813;15345482;19402213;16399855
P08427	181,184,241	R. norvegicus	12922978
P09057	110,122	R. norvegicus	11253164

P09117	5,174,214,223,343,357	R. norvegicus	17254026
P09605	73,116,313	R. norvegicus	15345482
P09951	353	R. norvegicus	18803298
P10608	132,141,209,219	R. norvegicus	16043170
P10860	53,170,193,289,319,439,451,458,464,528,539,550	R. norvegicus	11593016;19402213
P10888	44,82	R. norvegicus	15345482
P11345	38,170,364	R. norvegicus	10801894
P11442	285,337,377,608,731,754,883,899,900,908,921,10	R. norvegicus	16157014;17254026
P11598	96,1128,1237,1487,1574	R. norvegicus	18072209;19402213
P11884	67,95,100,115,278,445,454,467	R. norvegicus	18072209
P12346	398,475,487,499,504	R. norvegicus	15345482
P12839	87,104,114,330,430,447,448	R. norvegicus	17254026 ;19956584
P13596	149,358,383,802	R. norvegicus	17254026
P14141	561	R. norvegicus	11593016
P14480		R. norvegicus	15345482
P14604	310,344,396,422,440	R. norvegicus	15345482
P15651	112,	R. norvegicus	11593016
P15999	29,95,123	R. norvegicus	17254026
P16086	299,337,343,440	R. norvegicus	18803298
P16617	72,942,1261,2423	R. norvegicus	17254026;19402213
P17764	76,161,196,258	R. norvegicus	15345482
P18405	30,211,216,328,336	R. norvegicus	11593016
P18886		R. norvegicus	14641011
P19112	202,205,252,614,	R. norvegicus	11593016
P19511	58,280,287	R. norvegicus	15345482;17254026
P19527	50	R. norvegicus	16157014;17254026; 19956584
P21708	14,18,139,266,369,373	R. norvegicus	11593016
P22791	82,146,149,157	R. norvegicus	11593016
P23457	62,67,200,347	R. norvegicus	11593016
P23565	53,55,81,196	R. norvegicus	17254026;19956584
P23693	354,379,	R. norvegicus	15345482
P24329	27,134	R. norvegicus	11593016
P25113	48,99,108,262,272	R. norvegicus	18803298
P26255	4,26,50,92,119	R. norvegicus	16043170
P27791	142,211,221,333,343,360,392	R. norvegicus	17254026
P28480	70,307	R. norvegicus	16157014
P31596	121,131	R. norvegicus	20167432
P32551	538	R. norvegicus	15345482

P35355	54,70,73,75206,252,381,	R. norvegicus	9820830
P35571	134	R. norvegicus	19402213
P38918	218,219,239,246,440,480,487,501,628	R. norvegicus	18072209
P45592	107,141,164,168,191,252,268	R. norvegicus	17254026
P47819	68,117,140	R. norvegicus	16157014
P48500	114,347	R. norvegicus	17254026
P48675	48,165	R. norvegicus	15345482
P48679	82,387,404	R. norvegicus	11593016
P49950		R. norvegicus	15345482
P50137		R. norvegicus	11593016
P50398	58,83,137,141,147,150,173,275,309,321,363,447,	R. norvegicus	18803298
P50554	481	R. norvegicus	11593016
P55260	93,99,111,117,172,224,226,229,286	R. norvegicus	11593016
P59215	27,77,84,97	R. norvegicus	17254026
P60203	56,165,179,212,249,255	R. norvegicus	17254026
P60711	69,74,156,168,354	R. norvegicus	16284212
P61765	104,207	R. norvegicus	12124443
P62630	53,69,91,166,169,188,198,218,240,294,306,362	R. norvegicus	19402213
P62804		R. norvegicus	15345482
P63018	29,56,141,162,254,418	R. norvegicus	18072209
P63039	52,73,89,99	R. norvegicus	19402213
P63245	41,107,115,134,149,183,371,525	R. norvegicus	19402213
P63259	90,243,503	R. norvegicus	18072209
P85834	52	R. norvegicus	15345482
P85973	53,69,91,169,188,198,218,240,294,306,362	R. norvegicus	11593016
P97532	51,179,246,266,357	R. norvegicus	11593016
Q01986	50,88	R. norvegicus	10801894
Q02253	72,85,100,108,134,280	R. norvegicus	15345482
Q03336		R. norvegicus	11593016
Q05030	443	R. norvegicus	12682076
Q05962	14,113,194,219	R. norvegicus	15345482
Q06647	677	R. norvegicus	15345482
Q07439	81,95,112,132,187,191,195,251,291,297	R. norvegicus	18803298
Q07523	35,41,46	R. norvegicus	18072209
Q3KRE8		R. norvegicus	15345482
Q4KLZ6	103,111,130,277	R. norvegicus	17254026
Q4PP99	36,50,51,106,222,425	R. norvegicus	15345482
Q4QRB4	111,485	R. norvegicus	17254026

Q60587		R. norvegicus	15345482
Q62969	36,50,106,422	R. norvegicus	19118096
Q63632	301,332,337,343,403	R. norvegicus	18849345
Q63704		R. norvegicus	14641011
Q63787	28	R. norvegicus	15615788
Q64565	32,55,74,547,589	R. norvegicus	11593016
Q66HF1		R. norvegicus	15345482
Q6AYE4	13,79,102,182,195,411	R. norvegicus	15345482
Q6P6R2	596,636,695	R. norvegicus	15345482
Q6PDU7	111,289,353	R. norvegicus	15345482
Q9ER34	54,386,473	R. norvegicus	11593016;15345482;17254026;19402213
Q9JIR0	33	R. norvegicus	11923223
Q9QZ81	71,151,161,267,390,472	R. norvegicus	15345482
Q9R1Z0	95,1017,1020	R. norvegicus	19402213
Q9Z1E1	2,22,530,699,805,806,816,858	R. norvegicus	15934946
Q9Z1L0	7,22,48,62,118,195,225,247	R. norvegicus	15615788
Q9Z272	216,223,238	R. norvegicus	12058042
Q9Z2L0	246,256,405,425,962,1068	R. norvegicus	11593016;15345482;17254026;19402213
P22629	206,519	S. avidinii	8954558
P00360	7,22,118,153,195,225,247	S. cerevisiae	10746743
P00560		S. cerevisiae	1089655
P16622	47,54,75,253,274,312,316,318,328	S. cerevisiae	19695224
P19414	49,57,75,159,194	S. cerevisiae	19695224
P28241	132,137,146	S. cerevisiae	19695224
P38825	147,157,387	S. cerevisiae	19695224
P40513	37,157,186,190	S. cerevisiae	19695224
P47031	31,150,171,416,447,449	S. cerevisiae	19695224
P53318	228	S. cerevisiae	19695224
Q08968	351,677,705,709	S. cerevisiae	19695224
P05314	364	S. oleracea	19717529
O19067	511,534	S. scrofa	11099820
P00346	156,164,385,423,562	S. scrofa	728442
P10173	5,19	S. scrofa	6830817
Q29558	56,80,161,253,287	S. scrofa	7357008
Q64392	10,23,24,226,447	S. scrofa	14649731
Q8MHY0	245,249,543	S. scrofa	20386496
Q08276	9	S. tuberosum	19717529
Q43848	152	S. tuberosum	19717529

Q2PEP7	23,185,200,234,518	T. pretense	19717529
P01555	226,424723	V. cholerae	88548
P01556	265	V. cholera	88548
Q6F4I4	101,102,104,122	Z. elegans	19717529
P30920		B. circulans	8484906
P00669	48,66,151	B. Taurus	239931
P20488	73,83,145,666,670,671	B. Taurus	10428070
P02789	99,118,123	G. gallus	7092815
O14791	250,255,262	H. sapiens	2986587;8443247
P00441	91,101,111,112,243,258,419,434,450,540,543	H. sapiens.	11338199
P00918	230,273,351,354	H. sapiens.	16378731
P02461		H. sapiens.	19467351
P15121		H. sapiens.	14684358
P50552		H. sapiens.	18599602
Q02156	40,49,108,190,199	M. musculus	17255340
P00920	16,39,72	M. musculus	16622028
P02088	41,176,182,199,250,434	M. musculus	16622028
P08228	114	M. musculus	16622028
P14602	146	M. musculus	19150419
P20108		M. musculus	12821649
P23953		M. musculus	17615369
P24270	72,95,101,173	M. musculus	16622028
P39039	37,390	M. musculus	17615369
P51906	84,94,137,280,308,512	M. musculus	18093171
Q497L8		M. musculus	19150419
Q61012	97,98,502	M. musculus	16622028
Q64466		M. musculus	16622028
Q9QWK4		O. cuniculus	17615369
P10830		O. sativa	11839754
Q84YQ2	112,191	O. sativa	19717529
Q9FW84	41,176,182,199,250,433	R. norvegicus	19717529
O35077	183	R. norvegicus	18072209
O70351		R. norvegicus	19402213
P10111		R. norvegicus	17254026
P13233		R. norvegicus	17254026
P13437		R. norvegicus	11593016
P18090	277	R. norvegicus	16043170
P23928	20,182,198	R. norvegicus	15345482;19956584

P27139	356,366	R. norvegicus	18072209
P31399		R. norvegicus	15345482
P35434		R. norvegicus	15345482
P51635	86,115	R. norvegicus	18072209
P60881		R. norvegicus	12124443;17254026
P81155	35,41,50,140	R. norvegicus	19402213
Q64294		R. norvegicus	12922978
Q68FU3	130,259	R. norvegicus	15345482
Q6AYR0		A. cepa	11593016
Q9SDP1		A. thaliana	
O23223	234,249	A. thaliana	
O23715	117	A. thaliana	
O81147		A. thaliana	
Q9FFX7	26,149,158,160,198	A. thaliana	
Q9SEX2	27,75,152,161	B. cepacia	
P22088	178,233	B.	
P06278	109,330	licheniformis	
C4AU44	48,53,67,251,344	B. mallei	
Q9FEH8	106,468,509	B. napus	
O62698	72,77,83	B. taurus	
P00432	103,203,217,551,560,1249,1511,1626	B. taurus	
P00639	133,134	B. taurus	
P02584	84,94,274,280,308,512	B. taurus	
P04272		B. taurus	
P04409	7,25,129,140	B. taurus	
P05980	269,275	B. taurus	
P12344	195,395	B. taurus	
P23726	24,55,81,110,114,259	B. taurus	
P23727	67,75,96,179	B. taurus	
P42028	651	B. taurus	
Q3T165		B. taurus	
Q66LN0	145,154,211	B. taurus	
Q9XSC6	28,114,249	B. taurus	
P00800		B. taurus	
P00588	20,39,82	B.	
Q02307	62,103,338,342,528	thermoprote	
P02791	6,52,59,78,310,510,546	olyticus	
P00811	214,241,321	C. beta	

P00816	9	C.	
P03695	150	perfringens	
P23295		E. caballus	
P68139	84,92,115,137,387	E. coli	
O65352	387	E. coli	
P14208	55,71,93,281,296	E. phage	
	13,24,56,221	F. oxysporum	
	75,89,211	G. gallus	
		H. annuus	
		H. gammarus	

Table S5 Comparison of NTyroSite with existing predictors after peptide level redundancy condition using an independent test set.

Measurement	GPS-YNO2	iNitro-Tyr	NTyroSite
Sp	0.796	0.785	0.802
Sn	0.177	0.176	0.607
Pr	0.071	0.068	0.230
Ac	0.746	0.736	0.781
MCC	-0.011	-0.024	0.272

[a]The threshold values of GPS-YNO2 is considered as medium. However, the threshold value iNitro-Tyr is consistent with value defined in the server. Further, the proposed NTyroSite predictor threshold is controlled the same as at Sp 90% of training model performances.
